# A case of thoracic *SMARCA4*‐Deficient undifferentiated tumor successfully treated with combination Ipilimumab–Nivolumab

**DOI:** 10.1002/ccr3.6745

**Published:** 2022-12-13

**Authors:** Yoshio Nakano, Daisuke Sekinada, Yusuke Kuze, Norio Okamoto, Iwao Gohma, Yumiko Yasuhara

**Affiliations:** ^1^ Department of Respiratory Medicine Sakai City Medical Center Sakai City Japan; ^2^ Department of Pathology Sakai City Medical Center Sakai City Japan

**Keywords:** case report, *SMARCA4*, undifferentiated tumors

## Abstract

Thoracic *SMARCA4*‐deficient undifferentiated tumors are rare, with poor prognosis. A 73‐year‐old man presented to our hospital with dyspnea. Computed tomography‐guided biopsy revealed a *SMARCA4*‐deficient undifferentiated tumor. The patient was treated with combination ipilimumab‐nivolumab. The tumor reduced in size after two courses.

## Graphical abstract

The present case indicated that (1) combination ipilimumab–nivolumab successfully showed clinical benefits in the treatment of thoracic *SMARCA4*‐deficient undifferentiated tumors and (2) the good tolerability of combination ipilimumab–nivolumab.

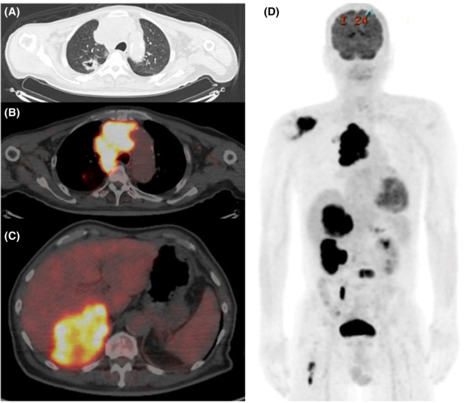

## 1 INTRODUCTION

Thoracic *SMARCA4*‐deficient undifferentiated tumors are high‐grade tumors of the thoracic region in adults, characterized by an anaplastic or rhabdoid phenotype and defective *SMARCA4* expression. These tumors occur more commonly in young to middle‐aged men with a history of heavy smoking and are often centered in the mediastinum or hilar regions. The prognosis is poor, with a median survival of 4–7 months due to poor response to treatment and high‐grade malignancy.^1^, ^2^ In general, cytotoxic chemotherapy is not effective; however, case reports of significant response to immune checkpoint inhibitors have been published.^3^, ^4^, ^5^, ^6^ Combination ipilimumab–nivolumab reportedly significantly prolongs overall survival (OS) compared with chemotherapy in patients with non‐small cell lung cancer,^7^ although no cases of *SMARCA4*‐deficient undifferentiated tumors treated with combination ipilimumab–nivolumab have been reported.

## 2 CASE PRESENTATION

A 73‐year‐old man with a 10 years history of diabetes and heart failure and a 50‐pack‐year smoking history underwent computed tomography (CT) in November of year X‐1 and was suspected to have lung cancer. Subsequently, the patient's respiratory distress worsened on approximately January X, and he visited our hospital on January 18th the same year, and the following vital signs were documented: oxygen saturation (SpO_2_, 95%); respiratory rate, 16 breaths/min; blood pressure, 110/83 mmHg; and heart rate, 122 beats/min. Endobronchial ultrasound‐guided transbronchial needle aspiration was performed on lymph nodes #7 and #4R with a 21‐gauge puncture needle, although the cytology results were inconclusive. CT‐guided biopsy was performed for metastatic lesions in the right adrenal gland with an 18‐gauge puncture needle. Histopathological examination revealed diffuse sheets of proliferating and highly atypical epithelioid cells with coagulation necrosis.

**FIGURE 1:**
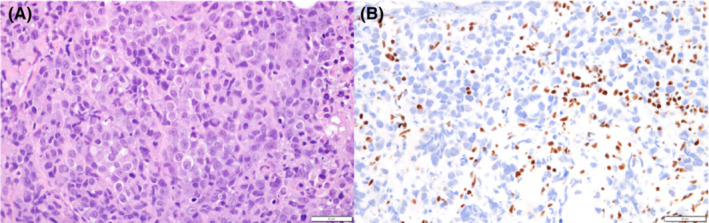
Histopathology of adrenal gland metastasis. A: The biopsy specimen shows diffuse sheets of proliferating and highly atypical, epithelioid cells with coagulation necrosis. The tumor cells are relatively monotonous, with eosinophilic cytoplasm, vesicular chromatin, 1–2 prominent nucleoli, and partial rhabdoid appearance (Hematoxylin–Eosin Stain, 10 × 40). B: The tumor cells completely lacked *SMARCA4* expression (SMARCA4 immunostaining, 10 × 40)

The tumor cells were relatively monotonous, with eosinophilic cytoplasm, vesicular chromatin, 1–2 prominent nucleoli, and a partial rhabdoid appearance (Figure 1).

**FIGURE 2:**
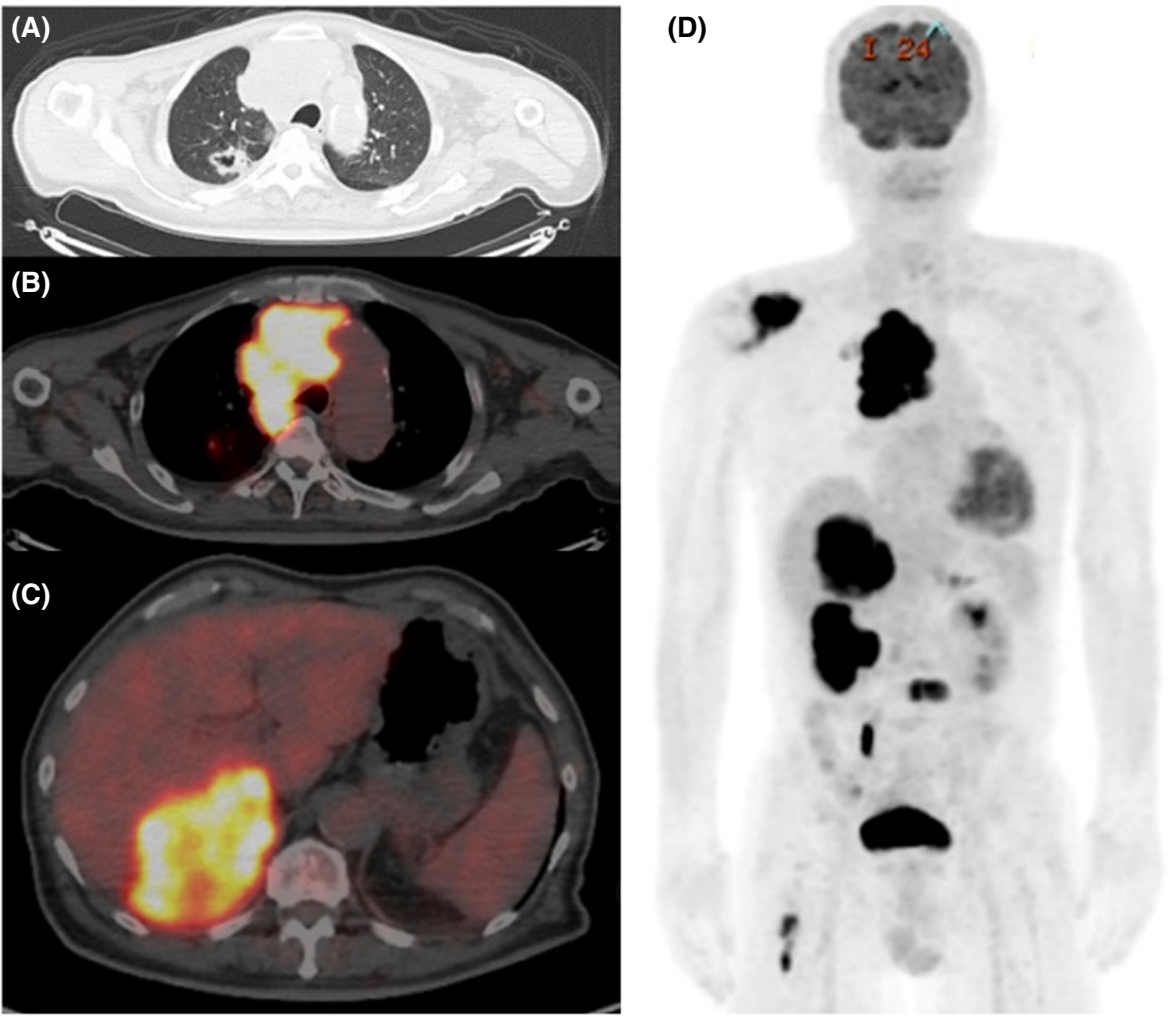
PET CT scan. (A) A 3.4 cm primary tumor in the right upper lobe; (B) mediastinal lymph node metastases; (C) large metastatic lesion in the right adrenal gland; and (D) multiple bone metastases. CT, computed tomography; PET, positron emission tomography

Immunohistochemically, the tumor cells totally lacked *SMARCA4* and BRM (*SMARCA2*); were negative for TTF1, AE1/3, Claudin4, desmin, MyoD1, myogenin, CD99, CD56, and WT‐1 proteins; and were weakly positive for synaptophysin. Based on these results, the tumor was classified as a thoracic *SMARCA4*‐deficient undifferentiated tumor. Driver gene screening showed negative results for *EGFR, ALK, ROS‐1, KRAS,* and *BRAF*. Immunohistochemical expression of programmed death‐ligand 1 (PD‐L1) was found to be 10%–20%. Positron emission tomography CT showed a 3.4 cm primary tumor in the right upper lobe of the lung and metastases to the mediastinal lymph nodes, multiple bones, and bilateral adrenal glands (Figure 2). The tumor was thus staged as clinical stage IVB (cT2aN2M1c).

**FIGURE 3:**
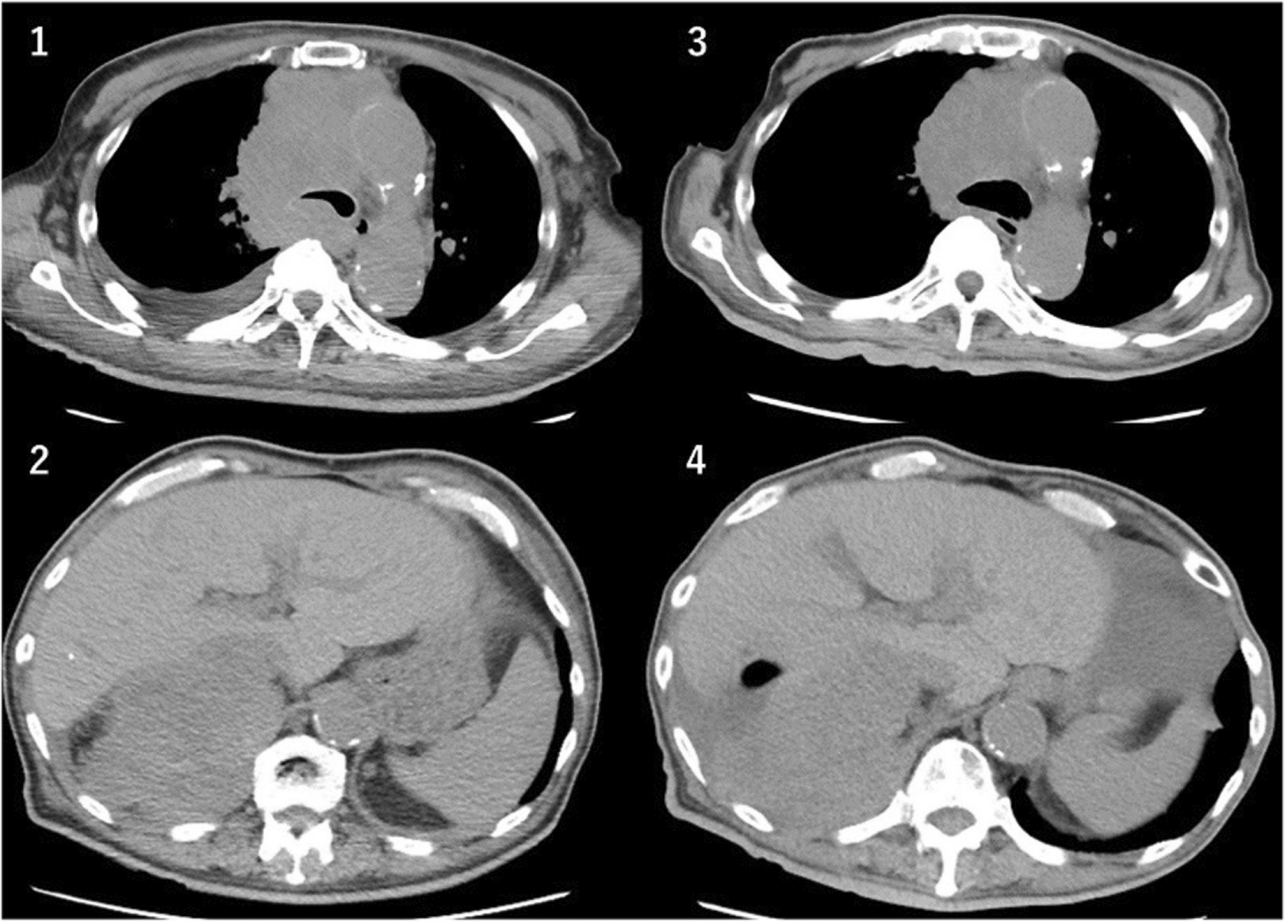
Comparison of pre‐ and post‐therapy CT scans. (1) Mediastinal lymph node metastases at the beginning of treatment; (2) right adrenal gland metastasis at the beginning of treatment; (3) mediastinal lymph node metastases at the end of the treatment second course; and (4) right adrenal gland metastasis at the end of the second treatment course

On February 22nd of year X, the patient's condition deteriorated, and he was admitted due to respiratory distress with an SpO_2_ of 88%. He was assigned performance status 2 (PS2) because of his inability to walk owing to right femoral bone metastasis. After obtaining informed consent, combination ipilimumab–nivolumab (nivolumab at a dose of 360 mg every 3 weeks plus ipilimumab at a dose of 1 mg/kg every 6 weeks) were initiated on February 24th of same year. Thereafter, home oxygen therapy was initiated, and combination ipilimumab–nivolumab was administered on an outpatient basis. On June 1st of the same year at the end of the second course, a repeat CT scan showed that the primary tumor and metastases had shrunk (Figure 3). The tumor progression was judged to have stabilized, according to the revised “Response Evaluation Criteria in Solid Tumors” guidelines (version 1.1).^8^

Clinically, the patient's dyspnea improved because the tumor had shrunk, and the tracheal stenosis had resolved. The patient had severe heart failure before the cancer diagnosis, and the echocardiographic evaluation revealed a decreased ejection fraction of 42.9% and decreased wall motion in all circumferential regions, especially from the septum to the anterior wall. On June 12th of same year, his underlying heart failure worsened, and he died suddenly. Our request to perform a clinical/pathological autopsy was denied by the patient's family. Because immune checkpoint inhibitor‐associated myocarditis is a rare adverse event, the incidence rate ranges between 0.27% and 1.14%,^9^ and our patient's chronic heart failure was severe enough to be the cause of death in this case, we opined that the worsening chronic heart failure was a result of its pre‐existing severity and was not directly related to the administration of combination ipilimumab‐nivolumab.

## 3 DISCUSSION

The present case highlights two clinical issues (3.1 and 3.2) related to combination ipilimumab–nivolumab therapy in a patient with a thoracic *SMARCA4*‐deficient undifferentiated tumor.

First, in our case, combination ipilimumab–nivolumab successfully showed clinical benefits in the treatment of thoracic *SMARCA4*‐deficient undifferentiated tumors (3.1). Thoracic *SMARCA4*‐deficient undifferentiated tumors are unresponsive to chemotherapy and have a poor prognosis, with a median life expectancy of 4–7 months.^1^, ^2^ Recently, case reports of these tumors responding to immune checkpoint inhibitors both alone (pembrolizumab^3^, ^4^ or nivolumab^6^) and in combination with chemotherapy (pembrolizumab plus carboplatin and pemetrexed,^10^ atezolizumab with bevacizumab, paclitaxel, and carboplatin^11^) have been published. However, no reports of response to ipilimumab–nivolumab combination exist. Only one case of combination therapy with two immune checkpoint inhibitors has been reported, in which ipilimumab was added some time after the onset of pembrolizumab administration.^12^ The Checkmate 227 study demonstrated that treatment with combination ipilimumab–nivolumab in patients with non‐small cell lung cancer was associated with a significant advantage in OS compared with chemotherapy, regardless of PD‐L1 expression. In the same study, 2 years OS tended to be better with combination ipilimumab–nivolumab than that with nivolumab alone in patients with PD‐L1 expression ≥1% (22C3) and ≥ 50%, respectively.^7^ In our case, the patient was able to receive combination ipilimumab–nivolumab without any apparent adverse events and had a successful response with symptom improvement. Unfortunately, the patient died from worsening chronic heart failure; however, we believe that a long‐term positive response might have been possible because the tumor had shrunk, and his respiratory distress symptoms and general condition had improved.

The second clinical issue is the good tolerability of combination ipilimumab–nivolumab (3.2). At the time of diagnosis, the patient was at PS2 (could not walk and required oxygenation,) making cytotoxic chemotherapy difficult to administer. In addition, combination ipilimumab–nivolumab was administered on an outpatient basis without any adverse events. The efficacy of PD‐1/PD‐L1 inhibitor monotherapy in the primary treatment of stage IV non‐small cell lung cancer in patients at PS2 is currently unclear, as the KEYNOTE‐024 and IMpower110 trials enrolled only patients at PS0–1 as eligibility criteria.^13^, ^14^ A study on pembrolizumab in patients at PS2 (PePS2 study) reported that pembrolizumab could be safely administered even in patients with PS2.^15^ Furthermore, actual clinical practice has shown that immunotherapy‐related adverse events (irAEs) are not frequent, with most of them being mild and manageable.^16^

Because the patient had a thoracic *SMARCA4*‐deficient undifferentiated tumor that was not expected to respond to chemotherapy, and his general condition was poor (PS2), we discussed the best supportive care or immunotherapy with him. After an intense discussion, we decided to use both ipilimumab and nivolumab instead of a single agent to achieve a better response. Further studies will be conducted in similar cases to investigate the use of ipilimumab–nivolumab combination.

## 4 CONCLUSION

Combination ipilimumab–nivolumab was successfully administered to a patient with thoracic *SMARCA4*‐deficient undifferentiated tumor. These findings may be further validated in trials when chemotherapy is not possible owing to patient or tumor factors.

## AUTHOR CONTRIBUTIONS

**Yoshio Nakano:** Data curation; writing – original draft; writing – review and editing. **Daisuke Sekinada:** Investigation; visualization. **Yusuke Kuze:** Writing – review and editing. **Norio Okamoto:** Supervision. **Iwao Gohma:** Supervision. **Yumiko Yasuhara:** Investigation; supervision.

## FUNDING INFORMATION

This research did not receive any specific grants from funding agencies in the public, commercial, or not‐for‐profit sectors.

## CONFLICT OF INTEREST

None.

## ETHICAL APPROVAL

We adhere to the journal's code of ethics.

## CONSENT

Written informed consent was obtained from the patient for the publication of this case report.

## Data Availability

Data will be made available on request.
